# Effects of nitrogen application on ammonium assimilation and microenvironment in the rhizosphere of drip-irrigated sunflower under plastic mulch

**DOI:** 10.3389/fmicb.2024.1390331

**Published:** 2024-05-22

**Authors:** Zhaonan Chi, Yuxin Li, Jiapeng Zhang, Min Hu, Yixuan Wu, Xueqin Fan, Zhen Li, Qingfeng Miao, Weiping Li

**Affiliations:** ^1^College of Water Conservancy and Civil Engineering, Inner Mongolia Agricultural University, Hohhot, China; ^2^Inner Mongolia Key Lab of Molecular Biology, Inner Mongolia Medical University, Hohhot, China; ^3^Vocational and Technical College of Inner Mongolia Agricultural University, Baotou, China

**Keywords:** ammonium assimilation, nitrogen, soil physicochemical properties, root exudates, functional gene

## Abstract

This study investigated the effect of nitrogen application on the rhizosphere soil microenvironment of sunflower and clarified the relationship between ammonium assimilation and the microenvironment. In a field experiment high (HN, 190 kg/hm^2^), medium (MN, 120 kg/hm^2^) and low nitrogen (CK, 50 kg/hm^2^) treatments were made to replicate plots of sunflowers using drip irrigation. Metagenomic sequencing was used to analyze the community structure and functional genes involved in the ammonium assimilation pathway in rhizosphere soil. The findings indicated that glnA and gltB played a crucial role in the ammonium assimilation pathway in sunflower rhizosphere soil, with Actinobacteria and Proteobacteria being the primary contributors. Compared with CK treatment, the relative abundance of Actinobacteria increased by 15.57% under MN treatment, while the relative abundance decreased at flowering and maturation stages. Conversely, the relative abundance of Proteobacteria was 28.57 and 61.26% higher in the MN treatment during anthesis and maturation period, respectively, compared with the CK. Furthermore, during the bud stage and anthesis, the abundance of Actinobacteria, Proteobacteria, and their dominant species were influenced mainly by rhizosphere soil EC, ammonium nitrogen (${\text{NH}}_{4}^{+}$-N), and nitrate nitrogen (${\text{NO}}_{3}^{-}$-N), whereas, at maturity, soil pH and ${\text{NO}}_{3}^{-}$-N played a more significant role in shaping the community of ammonium-assimilating microorganisms. The MN treatment increased the root length density, surface area density, and root volume density of sunflower at the bud, flowering, and maturity stages compared to the CK. Moreover, root exudates such as oxalate and malate were positively correlated with the dominant species of Actinobacteria and Proteobacteria during anthesis and the maturation period. Under drip irrigation, applying 120 kg/hm^2^ of nitrogen to sunflowers effectively promoted the community structure of ammonium-assimilating microorganisms in rhizosphere soil and had a positive influence on the rhizosphere soil microenvironment and sunflower root growth.

## 1 Introduction

Nitrogen is an indispensable nutrient for crop growth and thus a limiting factor in agricultural ecosystems (Man et al., 2020). However, the majority of nitrogen in the soil needs to be converted into absorbable ions through the nitrogen cycle (Kong et al., 2019; Rong et al., 2020). Ammonium assimilation is the process by which ion conversion occurs; specifically, ammonium-assimilating bacteria transform ammonia (NH_3_) or inorganic nitrogen compounds (like urea) into ammonium ions, thus regulating nitrogen supply and demand in the soil (Sieradzki et al., 2023). This process plays an important role in the balance of soil ecosystems. Consequently, the examination of microbial dynamics in relation to soil ammonium assimilation is of paramount importance for sustaining nutrient cycling in the soil and enhancing nitrogen utilization.

Studies have demonstrated that nitrogen application promotes nitrogen cycling genes, especially genes associated with assimilation (Enebe and Babalola, 2021). The key genes glnA, gltB, gltD, and gltS are crucial in converting ${\text{NH}}_{4}^{+}$-N into glutamate (GS) during microbial activity (Li et al., 2021). GS is a sensor of nitrogen metabolic state, and decreasing GS activity might uncouple ammonium assimilation from nitrogen fixation (Tang et al., 2023). Inhibition of the ammonium assimilation pathway promotes symbiont-like nitrogen-fixing microorganisms that reduce fixed nitrogen to ammonia and act on the crop (Patriarca et al., 2002; Udvardi and Poole, 2013). In the rhizosphere, crop roots, soil microbial communities and the soil environment interact (Surette et al., 2003), with crops influencing the composition of rhizosphere microbes through root exudates (Sun et al., 2021). Consequently, signaling substances such as organic acids and amino acids in root secretions change the rhizosphere environment and the plant's nutrient uptake, leading to enhanced uptake of nutrients such as nitrogen, magnesium and potassium, which ultimately impacts crop quality (Feng et al., 2021; Zhao et al., 2021; Seitz et al., 2023; Sun et al., 2023).

Rhizosphere microorganisms influence the development, nutrient uptake and stress response of crop plants through their metabolic activities (Zhang et al., 2019; Bai et al., 2022). Microbial community structure is established based on functional genes (Burke et al., 2011), and different species within the same group serve multiple functions (Herben and Goldberg, 2014). Using known functional genes involved in the ammonium assimilation pathway, we identified the community of species involved in this pathway (Zhang et al., 2022). Using metagenomic sequencing technology to determine gene composition and function within the nitrogen cycle, it is possible to explore gene-level connections between the ammonium assimilation pathway in rhizosphere soil and the soil environment.

While previous research has underscored the crucial role of soil microbes in key ecological processes, empirical studies examining the impact of ammonium-assimilating microorganisms on soil physicochemical properties and the rhizosphere microenvironment are scarce, highlighting the need for further research.

To address this gap, we conducted a sunflower drip irrigation experiment under plastic mulch, where we manipulated the rhizosphere soil microenvironment through different nitrogen application rates and quantified the relative abundance of key species and functional genes involved in the soil ammonium assimilation pathway using metagenomic sequencing, with the goal of elucidating: (1) the impact of nitrogen application on the rhizosphere soil microenvironment and root system during the reproductive growth period of sunflowers; (2) the interaction between ammonium-assimilating microorganisms and the rhizosphere soil microenvironment of sunflower during different reproductive growth periods. This study aimed to establish the ecological mechanisms for the ammonium assimilation process and the nitrogen cycle from a genomics perspective, providing theoretical support for better protection of the rhizosphere soil microenvironment and improved soil nitrogen utilization.

## 2 Materials and methods

### 2.1 Overview of the experimental area

The experiment took place from May to September 2022 at the Hailiutu Test Base at the Inner Mongolia Agricultural University, China. The base is situated on the Tumochuan Plain at 111°2′30^‴^E, 40°41′30″N, known for its flat terrain and fertile soil. The area experiences a temperate continental climate. During the experimental period, the average temperature was 20.7°C, and the total rainfall was 279.8 mm. The test site 0–20 cm layer of rhizosphere soil is sandy loam with an average bulk density of 1.72 g/cm3, average pH of 8.51. Based on the analysis of soil pH levels (Table 1), it is classified as mildly alkaline soil. The soil surface layer contains 15.89 g/kg organic matter, 99.31 mg/kg total nitrogen, 63.81 mg/kg hydrolysable nitrogen, 43.76 mg/kg available phosphorus and 216.28 mg/kg available potassium.

**Table 1:** Soil pH classification.

**pH value**	**Level**
<6.5	Acidity
6.5–7.5	Neutrality
7.5–8.5	Alkali
>8.5	Strong alkali

### 2.2 Experimental design

The edible sunflower variety HZ2399 was used in the experiment. Diamine phosphate was introduced with the plants at the time of sowing, using identical amounts across all treatments (18% N, which is equivalent to 50 kg/hm^2^ of pure N). Additionally, urea (46% N) was applied with irrigation at the bud stage, at three different levels: 140, 70, and 0 kg/hm^2^. This created three distinct N treatments, classified as high nitrogen (HN, 190 kg/hm^2^), medium nitrogen (MN, 120 kg/hm^2^) and a control with low nitrogen (CK, 50 kg/hm^2^). The irrigation system for each treatment remained consistent with 900 m3/hm^2^ of pressurized saline water being applied to the beds before sowing. Subsequently, drip irrigation under the plastic mulch was implemented during the reproductive period, with two instances of irrigation with a volume of 300 m^3^/hm^2^. Additional details on the experimental design can be found in Table 2.

**Table 2:** Experimental design.

**Treatment**	**Pure N applied/(kg**·**hm**^**−2**^**)**		**Total nitrogen applied/ (kg·hm^−2^)**	**Flooding quota/(m**3·**hm**^**−2**^**)**			**Irrigation quota/(m3·hm^−2^)**
	**Basic fertilization**	**Fertilizer at bud stage**		**Pressure salt water**	**Early bud stage**	**Late bud stage**	
HN	50	140	190	900	150	150	1,200
MN	50	70	120	900	150	150	1,200
CK	50	0	50	900	150	150	1,200

**Figure 1:**
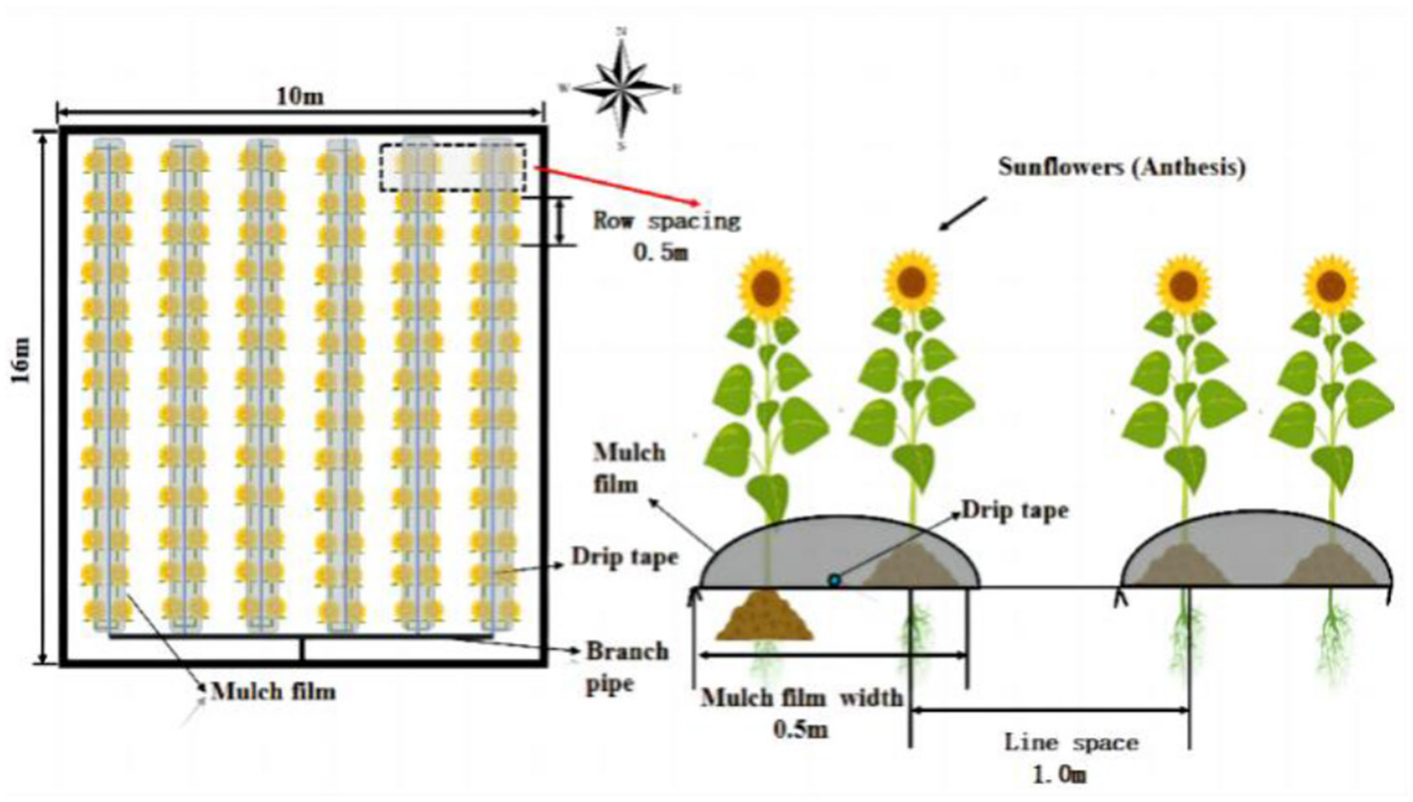
Layout of the test area.

The drip-irrigated sunflower system in this study used embedded patch-type drip tapes of 25 mm diameter, spaced 0.3 m apart, and flowing at a rate of 2 L/h. The experimental plot covered an area of 10 m × 16 m, surrounded by concrete barriers to prevent water, nitrogen and salt interactions between treatments. Sunflower seeds were sown on June 8, 2022 and seeds harvested on September 15. The planting pattern involved laying six films with 12 rows per plot, a row spacing of 50 cm and a plant spacing of 50 cm, resulting in a planting density of 21,000 plants per hectare. Each plot maintained a consistent planting density. The experiment was designed as a completely randomized block design, with each treatment replicated three times, totaling nine plots. Other field management practices were consistent with local cultivation habits. Additional details on the seeding design can be found in Figure 1.

### 2.3 Soil sample collection and processing

Soil samples were collected at different sunflower growth stages (bud stage, 55 days after sowing; anthesis, 65 days after sowing; maturation period, 105 days after sowing) using a root drill to sample 0–20 cm of each plant's rhizosphere soil. Samples were stored in sterile self-sealing bags, with mixed soil samples taken from three points within each plot. DNA extraction and macro-genome sequencing were performed on one subsample of each soil sample, while a second subsample was used to examine root secretion. The remaining subsample was dried naturally after passing through a 2 mm sieve and used to determine the soil's physical and chemical properties.

As per the test method outlined in the Chinese Environmental standard (HJ 802-2016), a soil extract using a soil-to-water mass ratio of 1:5 was employed to determine soil pH and electrical conductivity (EC) (Zhao et al., 2022). Soil EC and pH were measured in a soil-water extract with a ratio of 1:5 using a conductivity meter (DDS-307A, China) and pH meter (Mettler-Toledo, China). Ammonium nitrogen (${\text{NH}}_{4}^{+}$-N) in the soil was extracted with KCl (2 mol/L) and quantified using the indophenol blue colorimetric method. Nitrate nitrogen (${\text{NO}}_{3}^{-}$-N) was determined using a UV spectrophotometer after extraction with KCl (1 mg/L) in a soil-water ratio of 10:1.

Root exudates were extracted using an ethanol extraction method and analyzed using a GC-MS system (HP6890/5975C, USA). Chromatographic conditions included an HP-FFAP (30 m × 0.25 mm × 0.25 μm) capillary column, initial temperature 40°C (held for 2 min), ramped at 4°C/min to 220°C and held for 15 min; total run time 62 min. Injector temperature was 250°C; carrier gas was high-purity He (99.999%) with a column head pressure of 47.6 kPa and flow rate of 1.0 ml/min, non-split with a solvent delay time of 5 min. Mass spectrometry conditions: EI source; ion source temperature 230°C; quadrupole temperature 150°C. The relative content of each component was determined using the peak area normalization method.

### 2.4 Root collection and analysis

**Figure 2:**
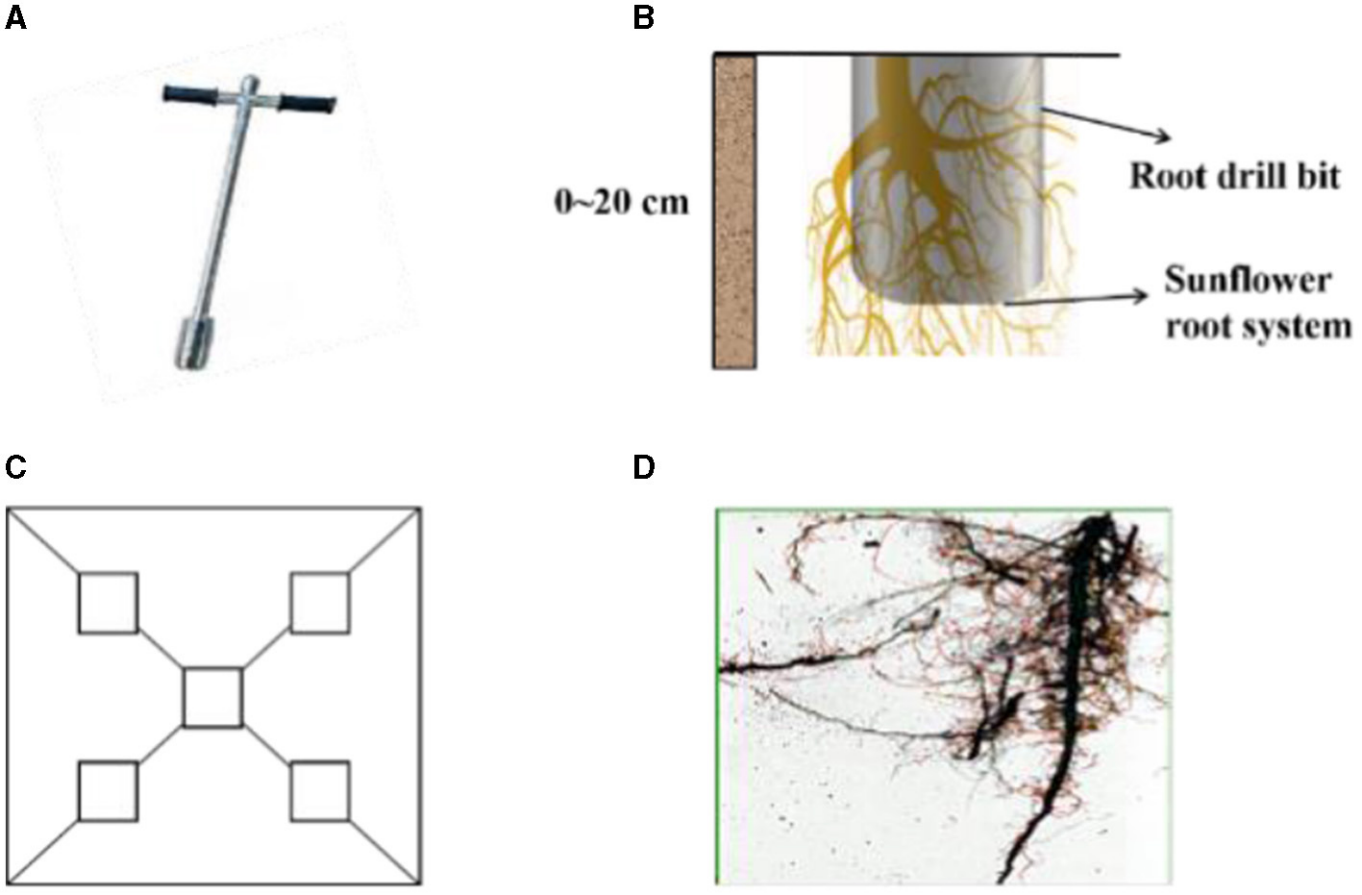
Root extraction process. **(A)** Root drill. **(B)** Root drilling to extract roots from 0–20 cm soil layer. **(C)** Root sampling location. **(D)** Root scanning images.

Root samples were collected at the bud stage, flowering stage and maturation stage from the 0–20 cm depth of sunflower plants using a root drill (Figure 2). Roots were cleaned with running water and then analyzed using a root analysis system (WinRHIZO) to obtain root length, root surface area and root volume (Figure 2D). Root length density (RLD) = root length (cm)/soil volume (cm^3^); Root surface area density (RSAD) = root surface area (cm^2^)/soil volume (cm^3^); root volume density (RVD) = root volume (cm^3^)/soil volume (cm^3^). Soil volume is defined as the quantity of soil obtained using a 15 cm tall and 9 cm wide cylindrical root drill in this experiment.

### 2.5 Metagenomic DNA extraction and shotgun sequencing

Approximately 500 mg of glass beads and 0.2–0.5 g of each soil sample were weighed and added to a 2 ml centrifuge tube along with 0.8 ml of Buffer SLXMlu. The mixture was vortexed for 1–5 min, followed by the addition of 80 μl Buffer DS before shaking and even mixing. The sample was then incubated at 70°C for 10 min, centrifuged at 13,000 × g at room temperature for 5 min and 200 μl of Buffer SP2 added. After shaking, 100 μl of HTR Reagent was added and the sample placed in an ice bath for 5 min. Post-centrifugation, 40 μl of magnetic beads were added and mixed evenly, followed by 450 μl of Binding Buffer. Subsequently, after liquid clarification, 500 μl of Binding Buffer, 1,000 μl of Buffer PHB, 1,000 μl of SPM Wash Buffer and 50 μl of Elution Buffer were added sequentially. Finally, total microbial genomic DNA was extracted using the OMEGA Mag-Bind Soil DNA Kit (M5635-02; Omega Bio-Tek, USA).

After extraction of genomic DNA, its quantification was achieved using a Qubit™ 4 Fluorometer (Qubit™ Assay Tubes: Q32856; Qubit™ 1X dsDNA HS Assay Kit: Q33231) (Invitrogen, USA) and agarose gel electrophoresis. Extracted microbial DNA was processed using an Illumina TruSeq Nano DNA LT Library Prep Kit for shotgun metagenomic library preparation. The preparation and sequencing of the shotgun metagenomic library were conducted by Pasinuo Technology Co., Ltd. (Shanghai, China) using the Illumina NovaSeq platform (Illumina PE150).

### 2.6 Metagenomic sequencing analysis

Raw sequencing reads were processed to obtain high-quality filtered reads for further analysis. First, sequencing adapters were removed from the reads using Cutadapt (v1.2.1). Next, low-quality reads were trimmed using a sliding-window algorithm in fastp. The high-quality filtered reads were then analyzed using the RefSeq-derived database Kraken2 (Wood et al., 2019). Subsequently, the ultra-large parameter set Megahit (v11.2) was used for assembly of each sample (Li et al., 2015). Overlapping clusters (exceeding 300 bp) were processed using mmseqs2 with the “easy-lincust” mode, setting the sequence identity threshold to 0.95, and covering residual of shorter overlapping groups to 90% (Steinegger and Söding, 2017). Clustering of CDS sequences from all samples was done using mmseqs2, setting the protein sequence identity threshold to 0.90, and covering the residual of shorter overlapping groups to 90%. Abundance values were normalized using CPM (copies per thousand bases per million mapped reads). Functional annotation of non-redundant genes was obtained using mmseqs2, targeting protein databases of KEGG, EggNOG and CAZy databases with search modes. EggNOG and GO annotations were done using the EggNOG-mapper (v2), obtaining GO ontology with map2slim and KO with KOBAS (Bu et al., 2021).

### 2.7 Statistical analysis

Each experiment was replicated three times. SPSS 25.0 (IBM, USA) was utilized for conducting single-factor ANOVA tests and Pearson correlation analyses. The ANOVA results were presented as mean ± standard deviation. Bar graphs of microbial community structure and heatmaps of soil microbial correlations in the rhizosphere were created using Origin 2021 (OriginLab, USA). Mantel test correlation analysis was performed using the vegan, ggcor, dplyr, and ggplot2 packages in R 4.2 (Alcatel-Lucent, USA) software developed. Redundancy analysis (RDA) of microbial metabolic functions and soil physicochemical factors was done using Canoco 5.0 (Microcomputer Power, USA).

## 3 Results

### 3.1 Effects of nitrogen application on physicochemical properties of sunflower rhizosphere soil

**Figure 3:**
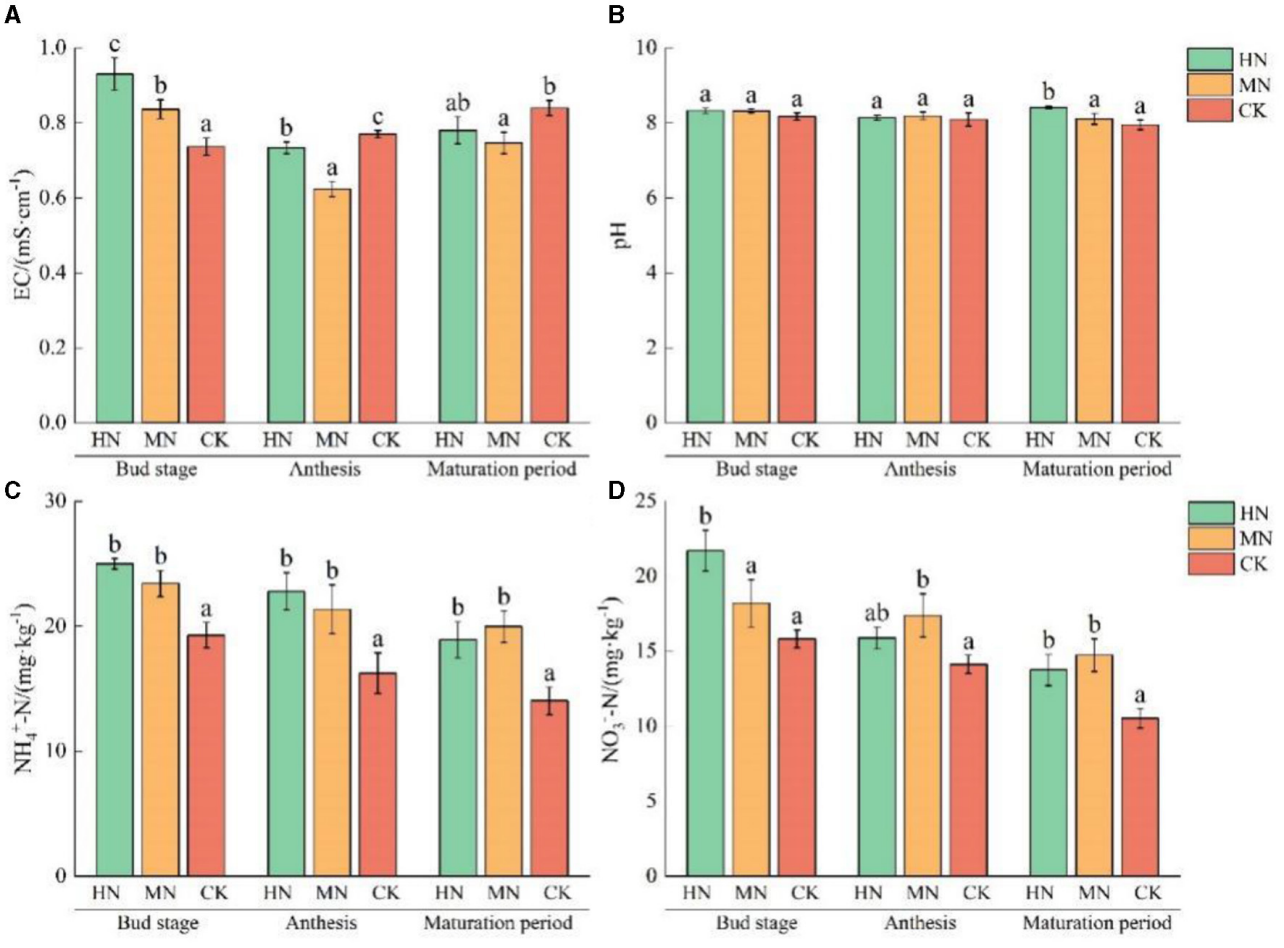
Effect of different nitrogen application treatments on soil physicochemical properties (*n* = 3). **(A)** EC, **(B)** pH, **(C)** ${\text{NH}}_{4}^{+}$-N, and **(D)** ${\text{NO}}_{3}^{-}$-N. Columns with different lowercase letters in the same fertility period are significantly different to each other at the 0.05 level. Total nitrogen applied: HN, 190 kg/hm^2^; MN, 120 kg/hm^2^; CK, 50 kg/hm^2^.

The effects of different soil nitrogen levels on physicochemical properties of rhizosphere soil are shown in Figure 3. Soil EC at the bud stage was 26.24 and 13.57% higher in the HN and MN treatments, respectively, compared with the control, CK (*P* < 0.05). During anthesis, soil EC was 4.77 and 19.05% lower in the HN and MN treatments, respectively, compared with CK (*P* < 0.05).

In rhizosphere soil, pH values during the maturation stage were 5.79 and 2.05% higher in the HN and MN treatments, respectively compared with CK. ${\text{NH}}_{4}^{+}$-N and ${\text{NO}}_{3}^{-}$-N content of rhizosphere soil gradually decreased with crop growth. ${\text{NH}}_{4}^{+}$-N and ${\text{NO}}_{3}^{-}$-N content were significantly higher in the HN and MN treatments than in the CK. Specifically, at the maturation stage, the ${\text{NH}}_{4}^{+}$-N content 34.96 and 42.50% higher in the HN and MN treatments than in the CK. The ${\text{NO}}_{3}^{-}$-N content increased by 30.74 and 40.13% in the HN and MN treatments, respectively, compared with the CK.

These results show that nitrogen application influences the physicochemical properties of the soil between roots, leading to an impact on the structure of nitrogen-cycling microbial communities in the soil.

### 3.2 Effects of nitrogen application on microbial species and genes involved in the ammonium assimilation pathway in rhizosphere soil

**Figure 4:**
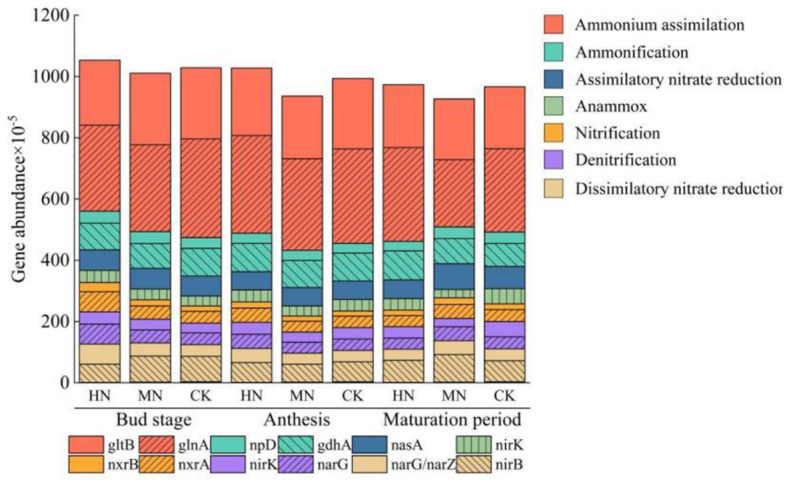
Relative abundance of key genes of nitrogen metabolism pathway (*n* = 3). Genes involved in the ammonium assimilation pathway: glnA, gltB; ammonification pathway genes: npD, gdhA; assimilatory nitrate reduction pathway genes: nasA; anammox pathway genes: nirK; nitrification pathway genes: nxrB, nxrA; denitrification pathway genes: narG, nirK; dissimilatory nitrate reduction pathway genes: nirB, narG/arZ.

By searching the macrogenomic database for genes related to the nitrogen cycle and choosing genes with >0.02% abundance in each pathway it can be seen that the ammonium assimilation pathway is a major route in the nitrogen cycle and the dominant genes are glnA and gltB (Figure 4). In the HN treatment, the abundance of glnA and gltB at the bud stage was 12.88 and 8.52% lower, respectively, compared with the CK. During anthesis in the MN treatment, the abundance of these genes was 3.21 and 11.00% lower, respectively, compared with the CK. However, at the maturation stage in the HN treatment, the abundance of glnA and gltB was 12.86 and 1.24% higher, respectively, than in the CK. Nitrogen application also increased the abundance of genes involved in assimilatory nitrate reduction, anammox ammonium oxidation, nitrification and denitrification pathways during the bud stage also increased in treatments with additional nitrogen compared with the control, while the abundance of ammonification genes was lower. In the MN treatment at the maturation stage, the abundance of genes involved in dissimilatory nitrate reduction, nitrification, assimilatory nitrate reduction and ammonification increased compared with the CK.

**Figure 5:**
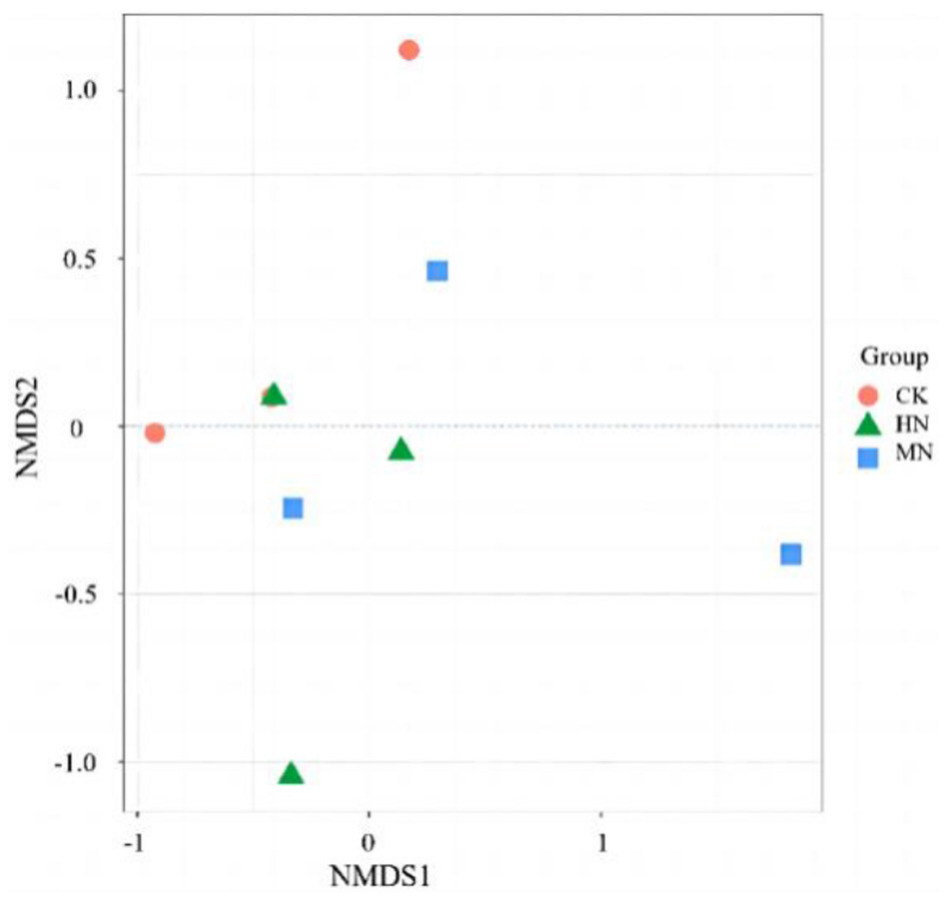
Rhizosphere soil microbial community structure under different nitrogen application treatments (*n* = 3). Total nitrogen applied: HN, 190 kg/hm^2^; MN, 120 kg/hm^2^; CK, 50 kg/hm^2^.

**Figure 6:**
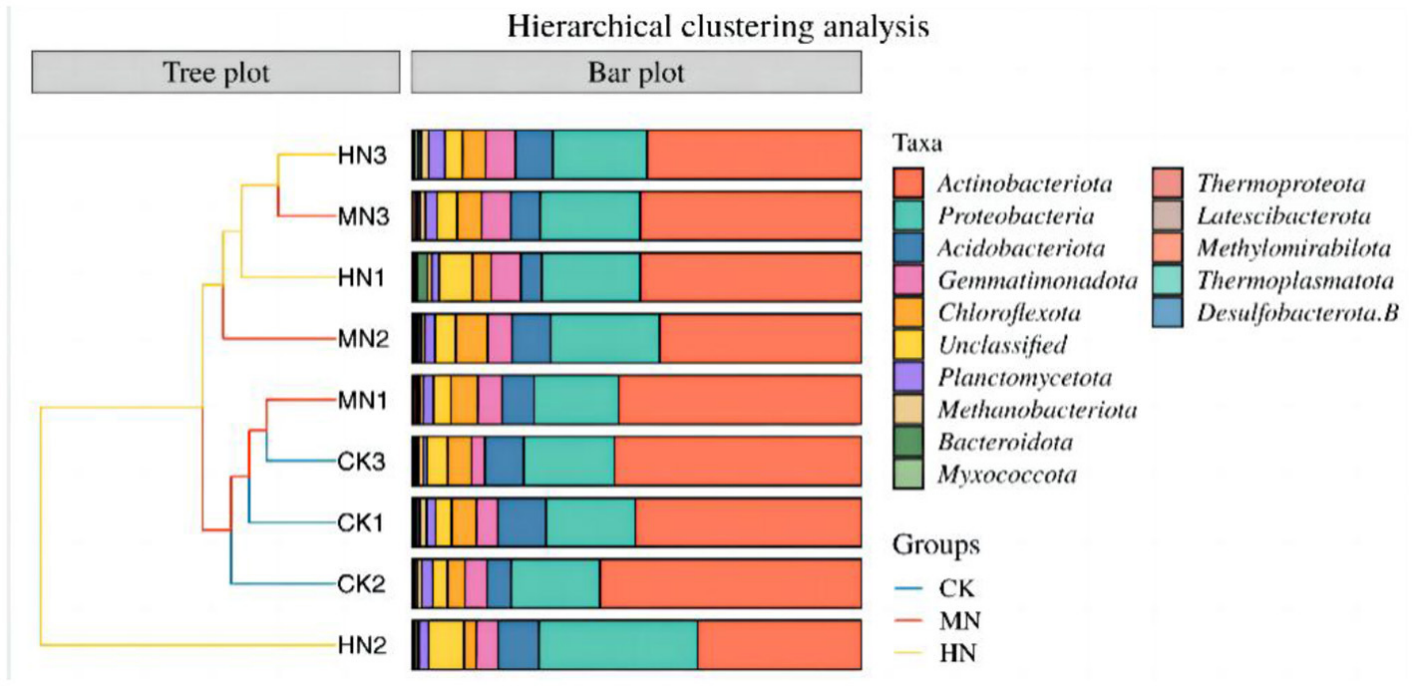
Relative abundance of phyla involved in ammonium assimilation under nitrogen application treatments (*n* = 3). CK1–CK3 are control treatments at the bud stage, anthesis and maturation period, MN1–MN3 are medium nitrogen treatments at the bud stage, anthesis and maturation period, and HN1–HN3 are high nitrogen treatments at the bud stage, anthesis and maturation period.

An NMDS analysis of rhizosphere soil microbial community structure based on the Jaccard distance algorithm revealed that in the HN and MN treatments, the abundance of microbial species involved in the nitrogen cycle were spatially distinct compared with the CK treatment, indicating significant differences in the composition of the rhizosphere soil microbial community due to nitrogen application (Figure 5). The microbial community structure in the HN, MN and CK treatments at the bud, anthesis and maturation stages were analyzed by clustering (Figure 6). Treatments were significantly clustered into three groups at each site at a threshold >0.06. CK replicates at the bud, anthesis and maturation stages were grouped with MN samples at the bud stage. Meanwhile, HN samples at the flowering period formed a separate group. Remaining samples from other periods were clustered into a single group. This clustering is visually represented in Figure 6.

In addition, Actinobacteria was the dominant phylum in this experiment, with Proteobacteria, Acidobacteria and Gemmatimonadota as major phyla. In comparison of fertility changes, the relative abundance of Actinobacteria phylum was found to be greater under HN and MN treatments during the stage of present buds as opposed to the maturation period and anthesis. Additionally, the relative abundance of Proteobacteria phylum and Acidobacteria phylum peaked under MN treatments during the anthesis stage, with levels reaching 0.24 and 0.09, respectively. Notably, during the bud stage, the relative abundance of Actinobacteria in the MN treatment was 7.26% greater than in the CK treatment. In contrast, during the bud stage, the relative abundance of Proteobacteria in the HN treatment was 10.66% higher than in the CK. Similarly, the relative abundance of Gemmatimonadota in the HN and MN treatments during the bud stage, increased by 37.39 and 13.20%, respectively, compared with the CK. These trends fluctuated during anthesis, where the relative abundance of Actinobacteria were 37.35 and 22.81% lower in the HN and MN treatments, respectively, while the relative abundance of Proteobacteria were 78.58 and 22.44% higher, respectively, compared with the CK. At maturity, the relative abundance of Actinobacteria in the HN and MN treatments were 13.29 and 10.56% lower, and the relative abundance of Proteobacteria were 4.14 and 10.54% higher, respectively, compared with the CK.

**Figure 7:**
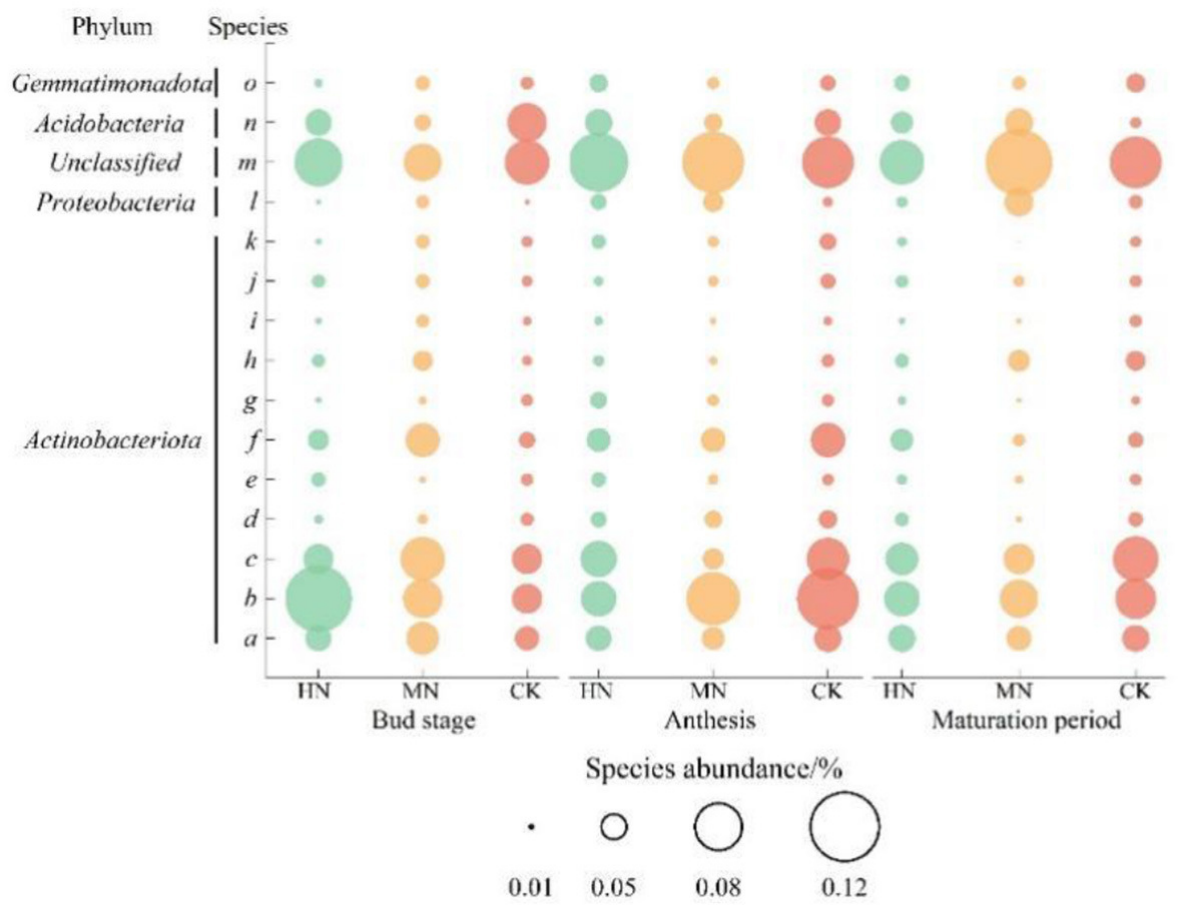
Abundance of species involved in ammonium assimilation under different nitrogen application treatments (*n* = 3). Species from the Actinobacteria phylum community: a (s_Unclassified_p_Actinobacteriota), b (s_Unclassified_g_VFJN01), c (s_Unclassified_g_Nocardioides), d (s_Unclassified_g_Solirubrobacter), e (s_Unclassified_f_Gaiellaceae), f (s_CADCTB01sp902805665), g (s_Unclassified_o_Solirubrobacterales), h (s_Unclassified_o_Acidimicrobiales), I (s_Unclassified_f_Nocardioidaceae), j (s_Unclassified_c_Actinomycetia), k (s_Unclassified_g_Aeromicrobium); Species from the Proteobacteria phylum community: l (s_Unclassified_g_Croceibacte–rium); Species from the Unclassified phylum community: m (s_Unclassified_d_Bacteria); Species from the Acidobacteriota phylum community: n (s_Unclassified_g_Gp6-AA40); Species from the Gemmatimonadota phylum community: o (s_Unclassified_f_Gemmatimonadaceae).

Statistical evaluation of the top 10 most abundant genes in the ammonium assimilation pathway identified 15 major species (Figure 7). The results showed that dominant species were from Actinobacteriota, unclassified or Acidobacteria such as a (s_Unclassified_p_Actinobacteriota), b (s_Unclassified_g_VFJN01), c (s_Unclassified_g_Nocardioides), m (s_Unclassified_d_Bacteria), n (s_Unclassified_g_Gp6-AA40); species l (s_Unclassified_g_Croceibacterium) in the Proteobacteria, and species o (s_Unclassified_f_Gemmatimonadaceae) from the Gemmatimonadota were major contributors. During the bud stage, the abundance of species a in the HN and MN treatments was 8.91 and 35.68% higher, respectively, than in CK, while species b was 121.05 and 30.00% higher, respectively, and species l was 31.75 and 56.83% lower, respectively. During anthesis, the abundances of species a, b and c in the HN and MN treatments were lower than in CK, but the abundances of species l and m were higher. During the maturation stage in the HN and MN treatments, the abundance of species b was 12.58 and 5.31% lower than in CK, while the abundance of species n was 95.18 and 147.73% higher, respectively. From the bud stage to maturation in the HN and MN treatments, the abundance of species a and c were initially lower than CK but then increased, species b was initially higher but then decreased, while species l and m were consistently higher than in CK.

### 3.3 Effect of nitrogen application on sunflower root system

**Figure 8:**
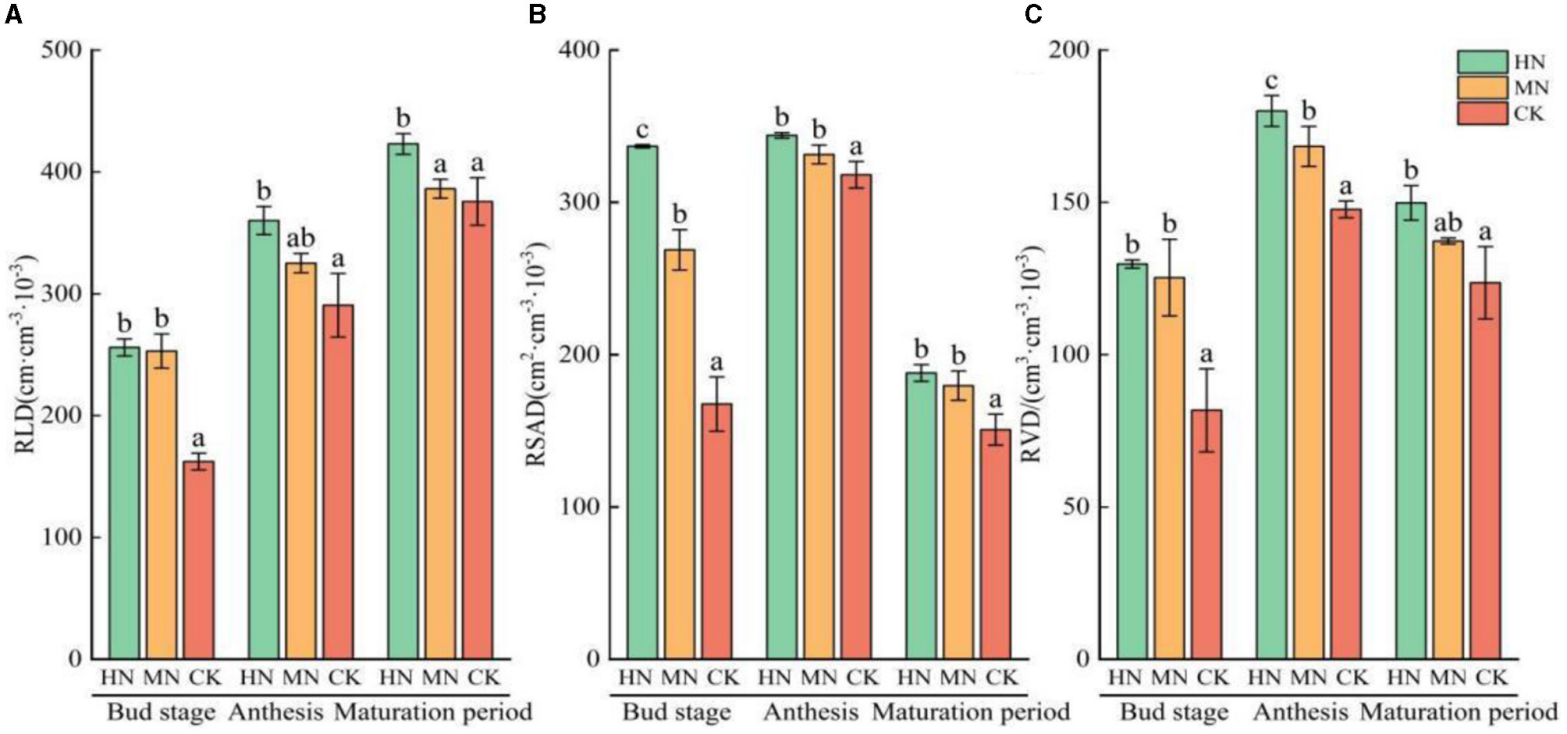
Root growth of sunflower under different nitrogen application treatments (*n* = 3). **(A)** RLD, **(B)** RSAD, **(C)** RVD. Columns with different lowercase letters in the same fertility period are significantly different to each other at the 0.05 level. Total nitrogen applied: HN, 190 kg/hm^2^; MN, 120 kg/hm^2^; CK, 50 kg/hm^2^.

Significant changes were observed in the growth of sunflower roots under different nitrogen levels (Figure 8). RLD, RSAD and RVD showed a gradual increase from the bud stage to anthesis, followed by a decrease from flowering to maturation. During the reproductive growth period, RLD, RSAD and RVD in both the HN and MN treatments were significantly higher than in the CK. Specifically, RLD in the HN treatment was 31.41% higher than in the CK, while RSAD and RVD were 44.62 and 33.94% higher, respectively, compared with the CK (*P* < 0.05). Similarly, in the MN treatment, RLD was 23.52%, 27.94% and 26.12% higher than the CK during the reproductive growth period; additionally, RSAD and RVD were 27.94 and 26.12% higher, respectively, compared with the CK.

Dominant organic acids in rhizosphere soil were oxalate, formate and malate (Table 3), with other major components including ascorbic acid, acetic acid and citrate. During the bud stage, the concentration of formate, ascorbic acid, acetic acid and citrate were significantly higher in the HN and MN treatments than in the CK, while oxalate concentration was 21.95 and 73.69% lower, respectively (*P* < 0.05). During the anthesis, the concentration of organic acids was significantly lower in the HN and MN treatments compared with CK, which achieved its maximum concentration. At maturation, the concentration of oxalate and malate was significantly higher in the HN and MN treatments compared with the CK, with oxalate concentration 300.52 and 186.56% higher than the control (*P* < 0.05); ascorbic acid and acetic acid concentrations were significantly lower under nitrogen application than in the control.

**Table 3:** Concentration of root secretion under nitrogen application treatment.

**Reproductive period**	**Treatment**	**Formate/μg g^−1^ DW**	**Malate/μg g^−1^ DW**	**Oxalate/μg g^−1^ DW**	**Ascorbic acid/μg g^−1^ DW**	**Acetic acid/μg g^−1^ DW**	**Citrate/μg g^−1^ DW**
Bud stage	HN	75.37 ± 4.57b	1,085.33 ± 40.70c	1,267.00 ± 60.92b	2.23 ± 1.07a	9.13 ± 0.47b	9.80 ± 1.31b
	MN	126.47 ± 5.01c	456.67 ± 35.50a	427.13 ± 22.91a	11.40 ± 0.98b	31.57 ± 2.64c	19.33 ± 2.61c
	CK	57.73 ± 5.20a	570.00 ± 60.06b	1,623.23 ± 55.47c	1.70 ± 0.17a	2.43 ± 0.25a	5.67 ± 0.95a
Anthesis	HN	100.57 ± 2.99a	916.00 ± 12.53b	1,514.67 ± 68.17b	1.50 ± 0.10a	8.53 ± 1.17a	14.57 ± 0.57a
	MN	97.60 ± 5.30a	614.33 ± 21.55a	1,135.03 ± 23.31a	1.67 ± 0.15a	8.73 ± 0.40a	12.80 ± 0.36a
	CK	159.33 ± 1.70b	1,017.00 ± 19.47c	2,409.43 ± 46.80c	6.43 ± 0.47b	63.63 ± 4.53b	30.83 ± 1.96b
Maturation period	HN	98.83 ± 3.59a	1,520.33 ± 34.78b	3,201.50 ± 43.76c	3.90 ± 0.50b	7.23 ± 0.12a	9.03 ± 0.31a
	MN	156.43 ± 33.99b	670.67 ± 119.00a	2,290.53 ± 95.71b	1.50 ± 0.17a	10.77 ± 0.57b	18.30 ± 6.61b
	CK	106.10 ± 6.08a	572.00 ± 74.51a	799.33 ± 47.52a	6.33 ± 1.53c	33.47 ± 1.27c	14.57 ± 0.93ab

The table presents data as means ± standard deviation (n = 3). For the same reproductive growth period data in the same column followed by different lowercase letters are significantly different to each other (P <0.05).

### 3.4 Correlation between phylum level microorganisms and rhizosphere microenvironment in ammonium assimilation

A linear analysis was conducted to examine the relationship between the relative abundance of dominant bacterial phyla, physicochemical properties and ammonium assimilation in sunflower rhizosphere soils. During the bud stage, a significant positive correlation was observed between the phylum Actinobacteria and pH, as well as ${\text{NH}}_{4}^{+}$-N. Additionally, phylum Proteobacteria exhibited a significant positive correlation with EC and ${\text{NO}}_{3}^{-}$-N, while phylum Gemmatimonadota showed a significant negative correlation with EC and ${\text{NO}}_{3}^{-}$-N. Conversely, the Unclassified phylum was significantly positively correlated with EC and ${\text{NO}}_{3}^{-}$-N (Supplementary Figure S1).

Moving to the anthesis stage, phylum Actinobacteria had a highly significant positive correlation with EC, and a significant negative correlation with ${\text{NH}}_{4}^{+}$-N and ${\text{NO}}_{3}^{-}$-N. Similarly, phylum Proteobacteria and Unclassified phylum both demonstrated significant negative correlations with EC, and significant positive correlations with ${\text{NH}}_{4}^{+}$-N and ${\text{NO}}_{3}^{-}$-N (Supplementary Figure S2).

In the maturation period, phylum Actinobacteria exhibited a significant positive correlation with EC, and a significant negative correlation with ${\text{NO}}_{3}^{-}$-N. Phylum Acidobacteria showed a significant negative correlation with EC, and a significant positive correlation with ${\text{NH}}_{4}^{+}$-N and ${\text{NO}}_{3}^{-}$-N. Furthermore, the Unclassified phylum displayed a significant positive correlation with pH (Supplementary Figure S3). These findings suggest that EC, ${\text{NH}}_{4}^{+}$-N, and ${\text{NO}}_{3}^{-}$-N were the primary factors influencing rhizosphere soil microorganisms during the bud stage and anthesis, while EC, pH, and ${\text{NO}}_{3}^{-}$-N were the main factors affecting microorganisms during the maturation period.

Pearson correlation analysis demonstrated significant associations between sunflower root secretions and the dominant phyla involved in ammonium assimilation at different growth stages (Supplementary Figure S4). Specifically, at the bud stage, Actinobacteria exhibited highly significant positive correlations with formalate, ascorbic acid, acetic acid and citric acid, while displaying highly significant negative correlations with oxalic acid. In contrast, Acidobacteria showed the opposite pattern, with highly significant negative correlations with formalate, ascorbic acid, acetic acid and citric acid, and highly significant positive correlations with oxalic acid. Moreover, phylum Proteobacteria and the unclassified phylum showed a highly significant positive correlation with malate. These findings highlight the influential role of Actinobacteria, Proteobacteria and the unclassified phyla in rhizosphere secretions at the bud stage.

Moving to the anthesis stage, phyla Actinobacteria and Proteobacteria displayed highly significant positive and negative correlations with six root secretions, respectively, further underscoring their impact on root secretions at this stage. Meanwhile, during the maturation period, phyla Actinobacteria and Gemmatimonadota showed highly significant negative correlations with formate and highly significant positive correlations with ascorbic acid. Additionally, phylum Proteobacteria demonstrated a highly significant positive correlation with formate and highly significant negative correlation with ascorbic acid. Notably, phylum Acidobacteria exhibited a highly significant positive correlation with oxalate, as well as a highly significant positive correlation with citric acid. The unclassified phylum showed a significant negative correlation with oxalate and malate and a significant positive correlation with citric acid. These findings contribute to our understanding of the complex interactions between sunflower root secretions and specific bacterial phyla at different growth stages.

### 3.5 Species and rhizosphere microenvironment correlations in ammonium assimilation

During the reproductive growth period of the sunflower, RDA analysis was used to examine major species in the ammonium assimilation pathway in relation to environmental factors in the rhizosphere (Supplementary Figure S5). The Actinobacteria phylum community's dominant species, namely s_Unclassified_p_Actinobacteriota, s_Unclassified_g_VFJN01 and s_Unclassified_g_Nocardioides, exhibited positive correlations with EC, pH and ${\text{NH}}_{4}^{+}$-N during the bud stage. However, during the anthesis period, the three species mentioned above exhibited negative correlations with pH, ${\text{NH}}_{4}^{+}$-N and ${\text{NO}}_{3}^{-}$-N; and with ${\text{NH}}_{4}^{+}$-N and ${\text{NO}}_{3}^{-}$-N during the maturation period. During anthesis and maturation periods, the dominant Proteobacteria species s_Unclassified_g_Croceibacterium and the undefined species s_Unclassified_d_Bacteria showed positive correlations with ${\text{NH}}_{4}^{+}$-N and ${\text{NO}}_{3}^{-}$-N, and negative correlations with EC.

During the maturation stage and the bud stage, the dominant species of Acidobacteriota phylum, s_Unclassified_g_Gp6-AA40, and the dominant species of Gemmatimonadota phylum, s_Unclassified_f_Gemmatimonadaceae, exhibited a negative correlation with soil pH, ${\text{NH}}_{4}^{+}$-N and ${\text{NO}}_{3}^{-}$-N. However, during the ripening stage, s_Unclassified_g_Gp6-AA40 showed a positive correlation with pH, ${\text{NH}}_{4}^{+}$-N and ${\text{NO}}_{3}^{-}$-N. Furthermore, according to significance analysis, EC, ${\text{NH}}_{4}^{+}$-N and ${\text{NO}}_{3}^{-}$-N were identified as the main factors influencing ammonium-assimilating species during the bud stage and anthesis, while pH and ${\text{NO}}_{3}^{-}$-N were the main factors influencing these species during the maturation period.

During the reproductive growth period, Spearman's correlation analysis was used to investigate the relationship between the dominant species of each ammonium assimilation pathway and the concentration of root secretion in the rhizosphere soil. The results revealed significant correlations (Supplementary Figure S6). Notably, the dominant species in the phyla Actinobacteria and Proteobacteria exhibited a significant negative correlation with oxalic acid only at the bud stage, while showing a significant positive correlation with other root secretions. Additionally, dominant species at the anthesis stage displayed a significant positive correlation with root secretions. Conversely, phylum Acidobacteria and the unclassified dominant species exhibited a significant negative correlation with rhizosphere secretions at the presenting stage and anthesis (*P* < 0.05). At the maturation period, phyla Actinobacteria and Gemmatimonadota were found to be positively correlated with acetic acid and ascorbic acid, and negatively correlated with formic acid. Conversely, species from the Proteobacteria, Acidobacteria phyla, and unclassified species showed a negative correlation with ascorbic acid while exhibiting a significant positive correlation with formic acid and citric acid.

## 4 Discussion

### 4.1 Effect of nitrogen application on physicochemical properties of sunflower rhizosphere soil

Nitrogen application directly influences the microenvironment of the rhizosphere, and soil physicochemical properties exhibit distinct patterns of change during the reproductive growth phase. Fertilizer can significantly increase the surface soil EC due to accumulation of excess nutrients in the soil, leading to accelerated salt accumulation in the 0–20 cm topsoil layer. Additionally, the H^+^ ions produced by ammonium assimilation exchange with the base ions adsorbed on the soil colloid surface (Sun et al., 2006), leading to an accumulation of H^+^ on the soil colloid surface and a decrease in soil pH. In this study, soil EC showed a decreasing trend from the bud stage to anthesis and then increased at maturation. The pH value consistently decreased from the bud stage to maturation (Figure 3), indicating that the decrease in soil pH is closely related to nitrogen decomposition and ammonium nitrogen assimilation.

In this study, urea was used as the nitrogen fertilizer, which upon decomposition increases the ammonium nitrogen content in the soil. Concurrently, nitrification driven by microbial communities increased nitrate nitrogen content. The content of ammonium and nitrate nitrogen decreased with crop growth, and ammonium nitrogen content was higher than nitrate nitrogen in this experimental area (Figure 3).

### 4.2 Effects of nitrogen application on gene expression and species in ammonium assimilation pathways

Through analysis of metagenomic sequencing results from 365 global sample points Nelson et al. (2016) found that the characteristics of nitrogen metabolism pathways are extremely similar. This study identified nitrogen metabolism pathways in rhizosphere soils, finding that the ammonium assimilation pathway was the most abundant nitrogen metabolism pathway (Figure 4). Based on KEGG analysis of gene functions, the most abundant genes in the GS and GOGAT families participating in ammonium assimilation were glnA and gltB. However, gene abundance decreased during the bud stage and anthesis under nitrogen application, indicating inhibition under nitrogen-rich (HN, MN) conditions and induction under nitrogen-limiting (CK) conditions, which is consistent with the findings of Stutz et al. (2007) and Enebe and Babalola (2021).

Actinobacteria and Proteobacteria play a significant role in ammonium assimilation (Wu et al., 2021). Nitrogen application at the budding stage enhances the presence of Actinobacteria, which stimulates the growth of ammonium assimilation microorganisms and elevates ${\text{NH}}_{4}^{+}$-N content. Research indicates that higher bacterial diversity can positively impact the capability of native microbes to defend against the intrusion of alien species (Shen et al., 2019; Zhang et al., 2022). Actinobacteria, the prominent phylum in the ammonium assimilation pathway, have the ability to resist diseases and safeguard plant health (Mitra et al., 2022). During the budding stage, the increase of the relative abundance of Actinobacteria can enhance sunflower root growth and help maintain a healthy rhizosphere. Consequently, the capacity to accumulate ${\text{NO}}_{3}^{-}$-N during the nitrification process also increased, providing a nutrient-rich environment for crops (Nannipieri et al., 2017).

Soil ${\text{NH}}_{4}^{+}$-N and ${\text{NO}}_{3}^{-}$-N are essential nutrients for plants, playing an important role at different growth stages (Both et al., 2019; Wu et al., 2021). During the bud stage and anthesis, rhizosphere soil EC, ${\text{NH}}_{4}^{+}$-N, and ${\text{NO}}_{3}^{-}$-N were identified as key factors influencing the phylum Actinobacteria and its related groups, including s_Unclassified_p_Actinobacteriota, s_Unclassified_g_VFJN01 and s_Unclassified_g_Nocardioides. Phylum Proteobacteria and its dominant species, s_Unclassified_g_Croceibacterium, and phylum Acidobacteria and its affiliated dominant species, s_Unclassified_g_Gp6-AA40, were also significantly impacted by these soil properties (Supplementary Figures S1–S3, S5). These findings emphasize the importance of soil ammonium assimilation capacity, with pH and ${\text{NO}}_{3}^{-}$-N identified as significant factors influencing the dominant phylum and species during maturation. Such insights could potentially be leveraged to enhance soil ammonium assimilation capacity in practice.

### 4.3 Relationship between ammonium assimilating microorganisms and the root system

Nitrate (${\text{NO}}_{3}^{-}$) and ammonium (${\text{NH}}_{4}^{+}$) are the primary inorganic nitrogen sources for crop growth and development (Hachiya and Sakakibara, 2017). In this experiment, the application of pure nitrogen at the bud stage of sunflower resulted in increased RLD, RSAD and RVD in comparison with no nitrogen application (CK; Figure 8). However, excessive assimilation of ${\text{NH}}_{4}^{+}$ as the sole or primary nitrogen source can reduce the carbon source available for plant growth (Coskun et al., 2013), thereby inhibiting root development (Britto and Kronzucker, 2002). In contrast, ${\text{NO}}_{3}^{-}$ typically does not exhibit toxicity symptoms in plants (Bittsánszky et al., 2015). Thus, assimilation through glnA and gltB into organically available nitrogen for plants plays a role in alleviating nitrogen deficiency through direct ${\text{NH}}_{4}^{+}$ absorption from the roots (Maeda et al., 2014). Thus, the sunflower rhizosphere soil in this experiment had a higher concentration of ammonium nitrogen compared with nitrate nitrogen, creating favorable conditions for root growth.

Plants secrete root exudates such as malate and citrates, facilitating uptake of mineral elements like P, Fe, Zn, Mn, and attract more beneficial bacteria to gather in the rhizosphere, which can trigger induced systemic resistance in plants (Lakshmanan et al., 2012). In this study, nitrogen application reduced oxalate concentration during the bud stage and increased citrate concentration, while increasing oxalate and malate concentrations at maturation (Table 3), enhancing crop salt tolerance.

Species involved in ammonium assimilation are correlated with root exudates. Dominant phyla such as Actinobacteria and Proteobacteria in this study showed significant correlations with root exudates, similar to findings in *Arabidopsis* by Huang et al. (2019) and Seitz et al. (2023). Jog et al. (2014) found in wheat that Actinobacteria can directly solubilize phosphate by producing malate, phytase and chitinase, thus promoting wheat growth. In this study, dominant species of Actinobacteria and Proteobacteria showed negative correlations with oxalate and malate and positive correlations with formate during the bud stage, but positive correlations with root exudates during anthesis (Supplementary Figure S6). This indicates that during the root growth stage from bud to anthesis, formate plays a crucial role in influencing symbiosis between plants and microorganisms and in developing potential plant defense mechanisms. Additionally, alterations in the relationship between oxalate and malate during anthesis, as well as species involved in ammonium assimilation, underscore the heightened significance of the rhizosphere soil microenvironment. This reaffirms the crucial role of organic acids in mediating interactions between plants and microorganisms (Blakley and Simpson, 1964; Lugtenberg et al., 2001; Lebeis et al., 2015; Yuan et al., 2015).

## 5 Conclusion

In comparing the application of 190 and 120 kg/hm^2^ of pure nitrogen decreased the rhizosphere soil EC at flowering and maturity in sunflower under drip irrigation and membrane, compared to applying 50 kg/hm^2^ of pure nitrogen. Additionally, it led to increased inter-rhizosphere soil pH, ${\text{NH}}_{4}^{+}$-N content, and ${\text{NO}}_{3}^{-}$-N content in the bud stage, anthesis and maturation period. During the bud stage and anthesis, soil EC, ${\text{NH}}_{4}^{+}$-N, and ${\text{NO}}_{3}^{-}$-N were found to promote microorganisms for ammonium assimilation in the soil nitrogen cycle in the rhizosphere. In addition, it was observed that pH and ${\text{NO}}_{3}^{-}$-N played a significant role in increasing soil ammonium assimilation capacity during the maturation period. Notably, The dominant species of the phylum Actinobacteria and Proteobacteria, s_Unclassified_p_Actinobacteriota, s_Unclassified_g_VFJN01, s_Unclassified_g_Nocardioides, s_Unclassified_g_ Croceibacterium were the main players in increasing the microbial ammonium assimilation capacity. During the reproductive growth period of sunflower, applying 120 kg/hm^2^ of pure nitrogen was found to enhance root RLD, RSAD, and RVD compared to the application of 50 kg/hm^2^ of pure nitrogen. Furthermore, there was a positive correlation between oxalate and malate with the dominant species of Actinobacteria and Proteobacteria. In order to improve the community structure of soil microorganisms in the ammonium assimilation pathway and create a better microenvironment for sunflower root growth, it is recommended to adopt a nitrogen application mode of 120 kg/hm^2^ for the Tumochuan Plain in the Inner Mongolia Autonomous Region of China.

## Data availability statement

The original contributions presented in the study are included in the article/Supplementary material, further inquiries can be directed to the corresponding author.

## Author contributions

ZC: Formal analysis, Software, Validation, Writing – original draft. YL: Data curation, Formal analysis, Software, Writing – original draft. JZ: Formal analysis, Investigation, Writing – review & editing. MH: Software, Investigation, Writing – original draft. YW: Validation, Software, Visualization, Writing – review & editing. XF: Software, Visualization, Writing – review & editing. ZL: Formal analysis, Investigation, Writing – review & editing. QM: Validation, Visualization, Writing – review & editing. WL: Conceptualization, Methodology, Data curation, Visualization, Writing – original draft, Writing – review & editing.
