# Supplementary material for: Effects of nitrogen application on ammonium assimilation and microenvironment in the rhizosphere of drip-irrigated sunflower under plastic mulch
Source: Front Microbiol. 2024 May 22;15:1390331. doi: 10.3389/fmicb.2024.1390331 (PMC11150556; doi:10.3389/fmicb.2024.1390331)
Supplement: Supplementary file 1 [file Data_Sheet_1.PDF]

Supplementary Material

1 Supplementary Figures

Supplementary Figures 1

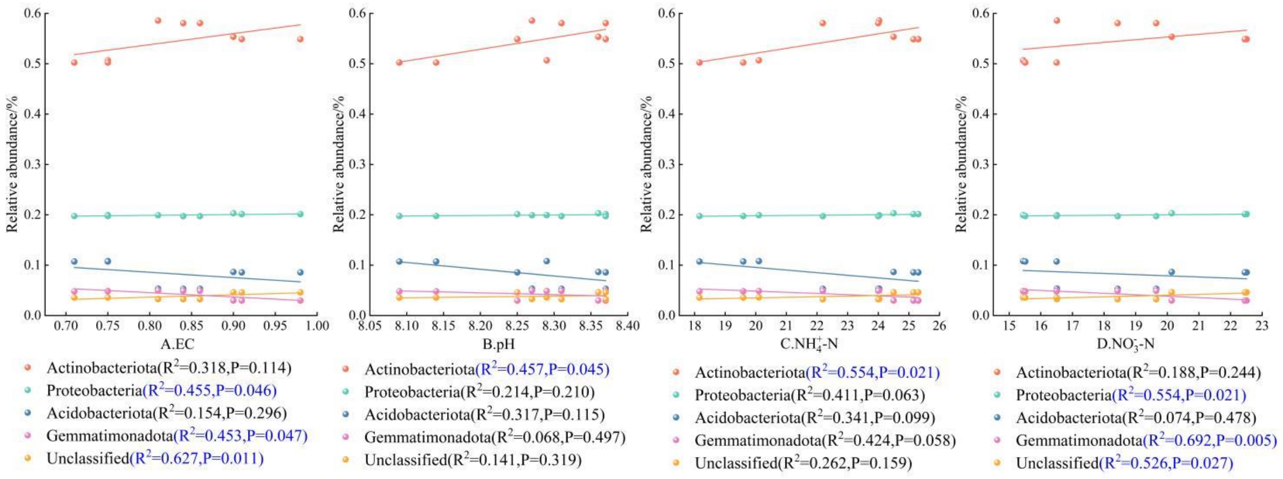

Supplementary Figure S1 Linear relationship between rhizosphere soil physicochemical properties and dominant bacterial phyla at the bud stage.

Supplementary Figures 2

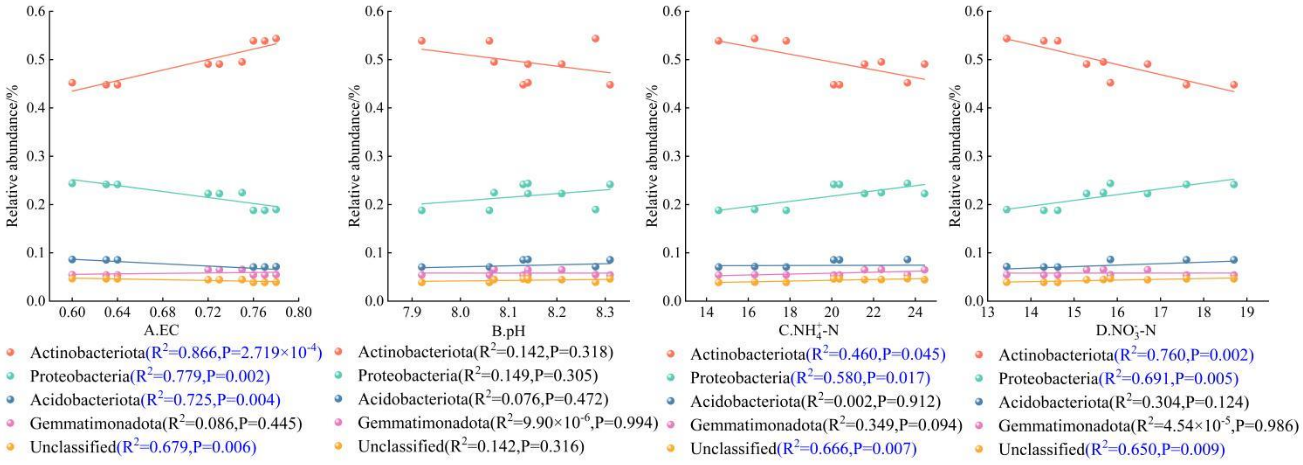

Supplementary Figure S2 Linear relationship between rhizosphere soil physicochemical properties and dominant bacterial phyla at anthesis.

Supplementary Figures 3

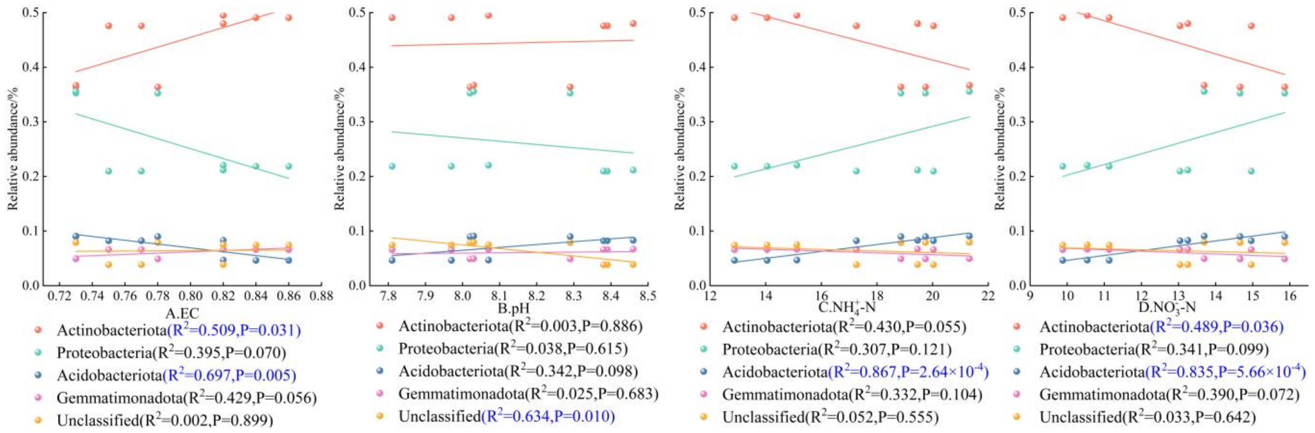

Supplementary Figure S3 Linear relationship between rhizosphere soil physicochemical properties and dominant bacterial phyla at maturation period.

Supplementary Figures 4

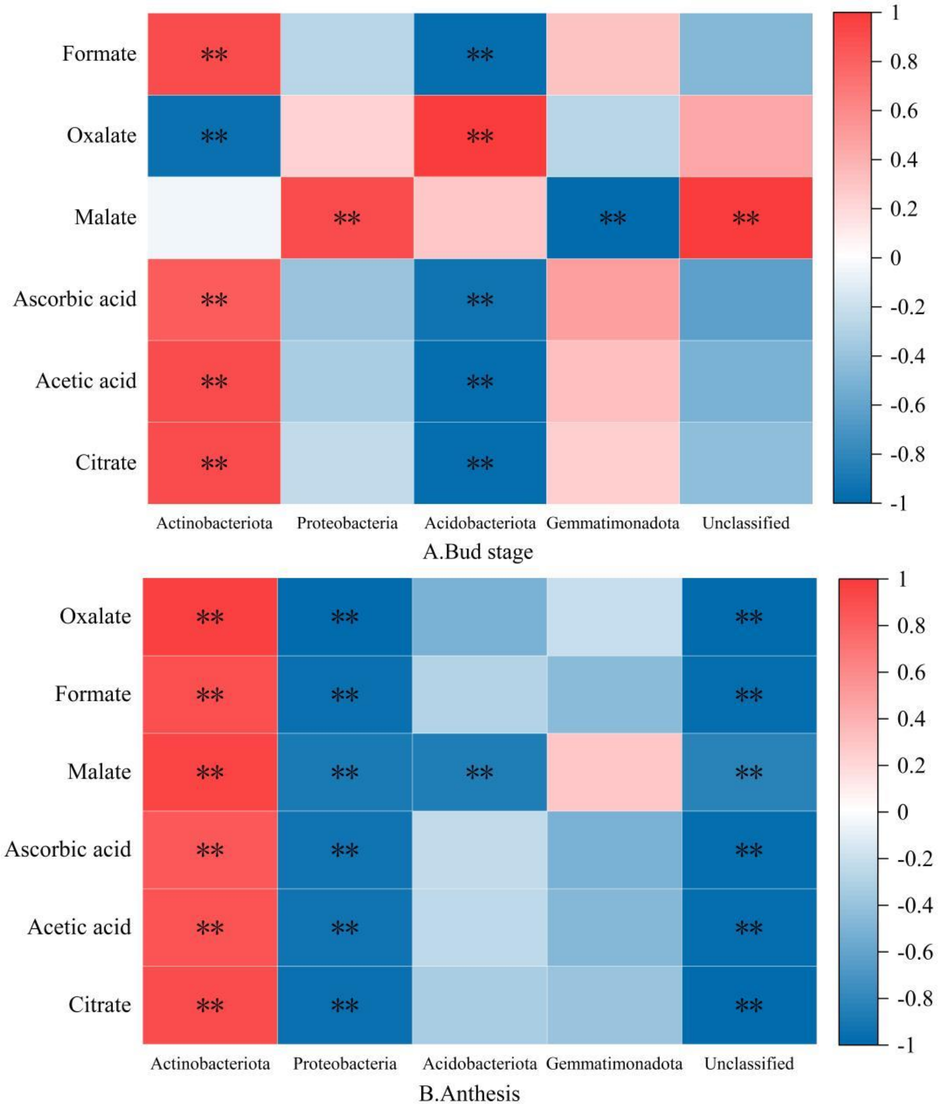

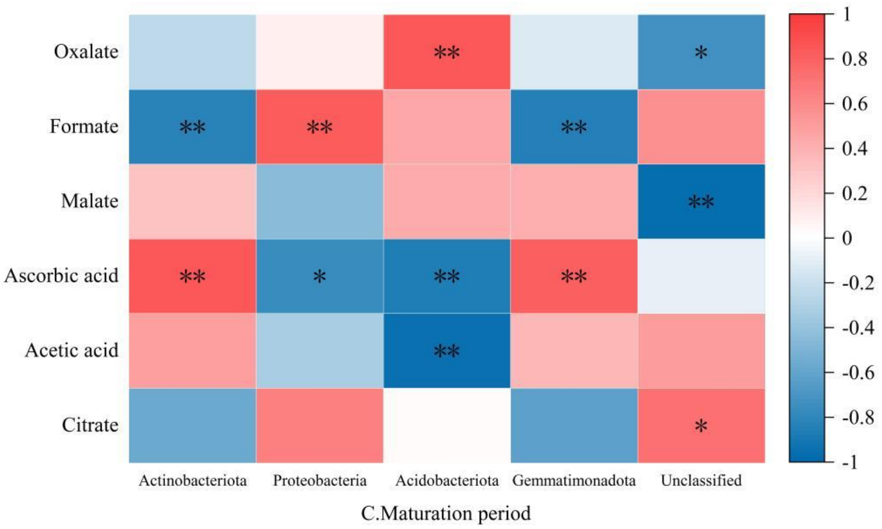

Supplementary Figure S4 Correlation of sunflower root secretions with dominant bacterial phyla

Supplementary Figures 5

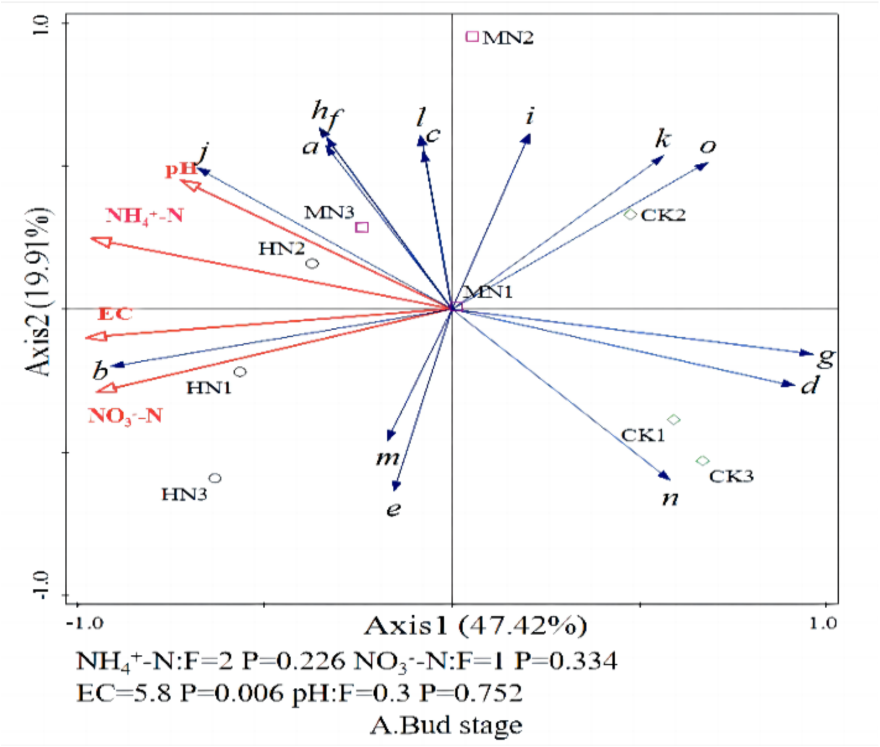

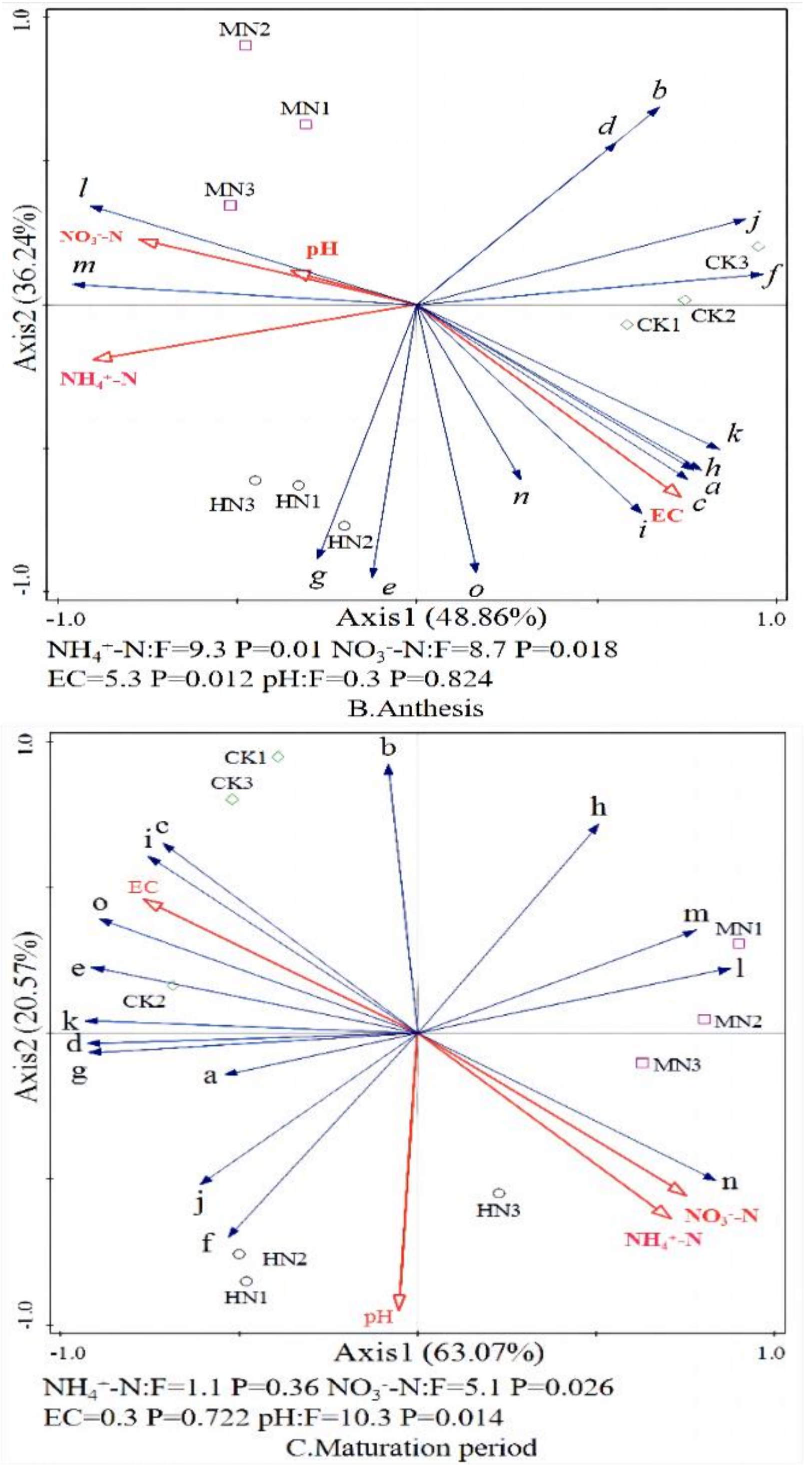

Supplementary Figure S5 Correlation between major species in the ammonium assimilation pathway and rhizosphere soil factors. Note: a~o: dominant species in the ammonium assimilation pathway, refer to Figure 7.

Supplementary Figure S6

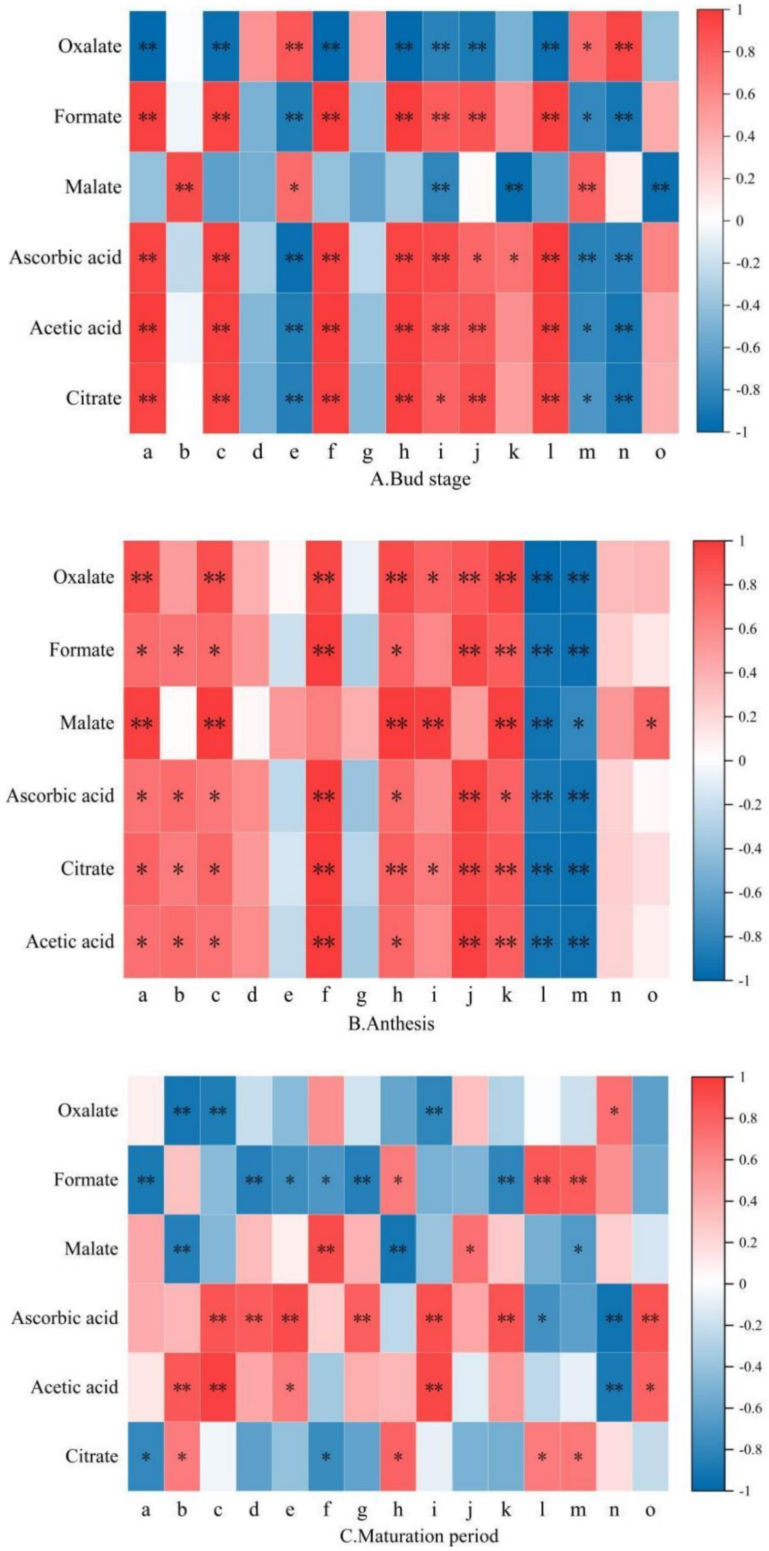

Supplementary Figure S6 Correlation of major species in the ammonium assimilation pathway with root secretion. Note: a~o: dominant species in the ammonium assimilation pathway, refer to Figure 7. * indicates P<0.05; **indicates P<0.01.
